# Learning curve comparison of robot-assisted and laparoscopic hepaticojejunostomy: a focus on critical suturing

**DOI:** 10.3389/fped.2025.1558362

**Published:** 2025-03-18

**Authors:** Jiahui Liu, Takuya Maeda, Chiyoe Shirota, Takahisa Tainaka, Wataru Sumida, Satoshi Makita, Yousuke Gohda, Yoichi Nakagawa, Aitaro Takimoto, Yaohui Guo, Daiki Kato, Akihiro Yasui, Akinari Hinoki, Hiroo Uchida

**Affiliations:** Department of Pediatric Surgery, Nagoya University Graduate School of Medicine, Nagoya, Japan

**Keywords:** hepaticojejunostomy, robot-assisted surgery, laparoscopic surgery, CUSUM analysis, congenital biliary dilatation, surgical approach

## Abstract

**Background:**

Robot-assisted surgery (RS) has gained popularity due to its potential advantages over conventional laparoscopic surgery (LS). However, the specific suturing steps that benefit most from RS in terms of efficiency remain unclear. This study aimed to compare the suturing performance and learning curves of RS and LS during hepaticojejunostomy.

**Methods:**

We retrospectively analyzed surgical videos of patients who underwent hepaticojejunostomy performed by the same surgeon between 2016 and 2023. Cases with incomplete data or conversion to open surgery were excluded. Suturing efficiency, anastomotic precision, and learning curves were evaluated using standardized metrics.

**Results:**

A total of 33 patients were included in the final analysis (17 RS, 16 LS). The median suture time per stitch was significantly shorter in the RS group (*P* = 0.017). The greatest efficiency gains were observed at the second (*P* = 0.041) and final stitches (*P* = 0.041). Complication rates were comparable between the two groups (*P* = 0.986).

**Conclusion:**

RS significantly improves efficiency at challenging suturing steps and provides a more consistent learning curve, highlighting its potential advantage for complex pediatric procedures such as hepaticojejunostomy. Future multicenter studies with larger sample sizes and longer follow-up are needed to validate these results and explore long-term outcomes.

## Introduction

Extrahepatic biliary resection and hepaticojejunostomy are the standard surgical treatments for congenital biliary dilatation (CBD) (1, 2). However, suture failure in hepaticojejunostomy remains a major concern, as it can lead to serious complications such as bile leakage, cholangitis, and anastomotic stenosis, which can prolong hospitalization and contribute to long-term issues like hepatolithiasis (3, 4).

Over the years, advances in surgical methods have led to the development of two primary techniques: conventional laparoscopic surgery (LS) and robot-assisted surgery (RS). RS has been increasingly utilized in complex biliary procedures due to its potential advantages in precision and dexterity (5, 6). Villegas et al. compared RS and LS in a porcine model and found that RS resulted in shorter anastomosis times and fewer suture failures. Moreover, they found that once surgeons were trained in laparoscopic vivo suturing, the learning curve for RS became significantly shorter (7).

Several retrospective studies comparing RS and LS in hepaticojejunostomy have also shown that RS significantly reduces operative time and anastomotic complications (8, 9). Furthermore, Leijte et al. demonstrated that the learning curve for RS in minimally invasive suturing is steeper than that of LS, indicating a faster adaptation to robotic techniques (10).

To objectively evaluate surgical proficiency and technical consistency, cumulative sum (CUSUM) analysis is a commonly used method to visualize learning curves. By tracking deviations from the mean performance time, CUSUM analysis allows for the identification of key proficiency milestones, making it particularly useful for assessing complex surgical steps in minimally invasive procedures (11). Previous studies have applied CUSUM analysis to robotic-assisted urethral and gastrointestinal anastomoses, demonstrating that robotic systems can significantly shorten the learning phase and stabilize performance (12, 13). However, existing research on hepaticojejunostomy has primarily focused on overall anastomosis time rather than specific suturing steps, leaving a gap in understanding how RS enhances precision at different stages of the procedure.

Unlike previous research, this study provides a detailed analysis of specific suturing positions, particularly the second and final stitches, which are among the most technically challenging steps in hepaticojejunostomy. By focusing on these key suturing points, we aim to provide new insights into how RS stabilizes surgical performance and enhances precision in complex suturing tasks.

Thus, the purpose of this study is to comprehensively compare RS and LS in hepaticojejunostomy by analyzing performance at specific suture sites. A better understanding of these differences may guide clinical decision-making and improve surgical training in minimally invasive pediatric surgery.

## Materials and methods

### Patient selection

This retrospective study included pediatric and adult patients with CBD who underwent RS or LS hepaticojejunostomy at Nagoya University Hospital between November 2016 and July 2023. All procedures were performed by a single board-certified surgeon. Patients with incomplete data or those requiring conversion to open surgery were excluded from the final analysis.

### Surgical method

All procedures were performed by the same surgeon following a standardized surgical protocol. RS and LS were performed using similar steps, differing only in port placements. In both groups, hepaticojejunostomy was performed using interrupted 5–0 absorbable sutures. In the LS group, electrocautery was used for dissection around the bile duct using monopolar scissors. In the RS group, monopolar scissors and bipolar Maryland forceps were used for dissection (14).

### Suturing method and data measurement

The hepaticojejunostomy was performed in a standardized manner:

1. Posterior wall suturing was completed first, starting from the right edge and proceeding leftward.
2. The final stitch of the posterior wall was placed at the leftmost edge.
3. The anterior wall suturing was then performed in the same manner, ensuring completion of the anastomosis.

Measurement of operative parameters:

1. Suture time per stitch: Defined as the time from the surgeon holding the needle to the completion of knotting.
2. Anastomotic diameter: Measured intraoperatively or from surgical videos, using the diameter of the forceps as a reference. The anastomotic diameter was defined as the distance between the right and left ends of the hilar bile duct.
3. Suture pitch: Measured intraoperatively using video analysis, with the forceps diameter as a reference

### Surgical video evaluation and an analysis of suture precision

The surgical videos were reviewed by the primary author, who independently assessed the suturing process. To ensure consistency and minimize bias, all videos were analyzed under identical viewing conditions, without patient identifiers or surgical outcome information. Postoperative bile leakage and cholangitis was used as an indicator of suture precision.

### CUSUM analysis

Cumulative sum (CUSUM) analysis was performed to assess the learning curve of robot-assisted surgery (RS) and laparoscopic surgery (LS) in hepaticojejunostomy. CUSUM analysis detects sequential changes in procedural performance by tracking deviations in anastomosis time from the group mean, allowing identification of the proficiency threshold.

The CUSUM score for each case was calculated as follows:

$${\mathrm{CUSUM}}_{n}=\;{\mathrm{CUSUM}}_{\left\{n-1\right\}}+\;(X_{n}-\;X_{\left\{\mathrm{mean}\right\}})$$

Where X_*n*_ represents the anastomosis time for case *n*, and X_mean_ is the overall mean anastomosis time across all cases.

The learning curve was divided into two phases:

Phase I (learning phase): Characterized by a continuous increase in the CUSUM curve, indicating the surgeon's skill acquisition and adaptation to the procedure.

Phase II (proficiency phase): Defined as the point where the CUSUM curve reaches its first major peak, followed by a sustained decline, indicating stabilization of performance.

Instead of applying a predefined statistical threshold, we determined the proficiency threshold based on the first major peak in the CUSUM curve, followed by a sustained decline, marking the transition from Phase I to Phase II.

### Statistical analysis

Categorical variables were described as frequencies and percentages, while continuous variables were reported as medians with interquartile ranges. Mann–Whitney *U*-tests were used to compare continuous variables, with statistical significance defined as *P* < 0.05.

Statistical analyses were performed using SPSS version 29.0.2.0 (IBM Corp., Armonk, NY, USA).

## Compliance with ethical standards

All procedures in this study complied with the ethical standards of the institutional and national research committees and the 1964 Declaration of Helsinki. This study was approved by the Ethics Review Board of Nagoya University Graduate School of Medicine (approval number: 2022-0474).

## Results

### Patient characteristics

A total of 34 patients were initially enrolled, but one RS case was converted to open surgery due to duodenal diverticulum perforation and was excluded, leaving 33 cases for analysis (17 RS, 16 LS). All LS cases were completed successfully.

Baseline characteristics, including age, sex, weight, and Todani classification, were comparable between the RS and LS groups, with no statistically significant differences (Table 1).

**Table 1:** Patient characteristics according to surgical approach.

Characteristic	RS (*n* = 17)	LS (*n* = 16)	*P*-value
Age (years)	14 (2–25.5)	7 (0.25–23.25)	0.402
Sex, Male/Female (%)	9 (53%)/8 (47%)	4 (25%)/12 (75%)	0.179
Body Weight (kg)	38.9 (9.6–53.3)	25.6 (7.14–48.98)	0.310

Todani classification (n) Ia: 4, Ic: 1, Iva: 9, Ia: 5, Ic: 3, Iva: 7, 0.986.

No dilatation: 3 No dilatation: 1.

Data are presented as medians (ranges).

RS, robot-assisted surgery for hepaticojejunostomy; LS, laparoscopic surgery for hepaticojejunostomy.

### Anastomosis characteristics and complications

The anastomotic diameter was significantly larger in the RS group [10 (7.85-12.9) mm] than in the LS group [7.3 (5.78–9.78) mm, *P* = 0.037]. Similarly, the RS group used more suture needles (median 14 vs. 11, *P* < 0.001), whereas suture pitch was comparable between the groups (*P* = 0.846).

Suture time per stitch was significantly shorter in the RS group (179.5 s vs. 201.75 s, *P* = 0.017), indicating improved efficiency in robotic suturing.

The complication rates were comparable between groups (RS: 11.8% vs. LS: 12.4%, *P* = 0.986), with bile leakage and cholangitis observed in both groups at similar rates (Table 2).

**Table 2:** Anastomosis characteristics according to surgical approach.

Characteristic	RS (*n* = 17)	LS (*n* = 16)	*P*-Value
Anastomosis Diameter (mm)	10 (7.85–12.9)	7.3 (5.78–9.78)	**0.037** *
Suture pitch (mm)	0.64 (0.57–0.84)	0.68 (0.52–0.91)	0.846
Number of sutures	14 (13–17)	11 (10–12.75)	**<0.001** *
Suture time per stitch(s)	179.5 (162.1–196.7)	201.75 (182.5–223.4)	**0.017** *
Related complications (%)	11.8% (2/17)	12.4% (2/16)	0.986
Bile leakage	1	1	–
Cholangitis	1	1	–

Data are presented as medians (ranges).

RS, robot-assisted surgery for hepaticojejunostomy; LS, laparoscopic surgery for.

hepaticojejunostomy.

Bold values with an asterisk (*) indicate statistically significant results (*P* < 0.05).

### Anastomosis time at Key suturing positions

Suturing times at key positions were compared (Table 3):

**Table 3:** Comparison of anastomosis time between each stitch according to surgical approach.

Stitch	RS (s)	LS (s)	*P*-value
1st (Right edge)	278 (217.5–318.5)	255.5 (218.75–358.5)	–
2nd	181 (164.5–233.0)	240.5 (187.0–292.75)	**0**.041*
Left edge	182 (150.5–213.5)	165 (151.25–230)	0.763
Last stitch	148 (127–172)	197 (140.75–250.5)	**0**.041*

Data are presented as medians (ranges).

RS, robot-assisted surgery for hepaticojejunostomy; LS, laparoscopic surgery for hepaticojejunostomy.

Bold values with an asterisk (*) indicate statistically significant results (*P* < 0.05).

Right edge stitch: No significant difference between RS and LS groups (278 s vs. 255.5 s, *P* = 1).

Second stitch: The RS group was significantly faster (181 s vs. 240.5 s, *P* = 0.041).

Left edge stitch: No significant difference was noted (182 s vs. 165 s, *P* = 0.763).

Last stitch: The RS group was significantly faster than the LS group (148 s vs. 197 s, *P* = 0.041).

These findings suggest that robotic assistance improves efficiency, particularly in complex suturing steps (second and last stitches), while maintaining consistency in simpler steps.

### Learning curve analysis (CUSUM analysis)

**Figure 1:**
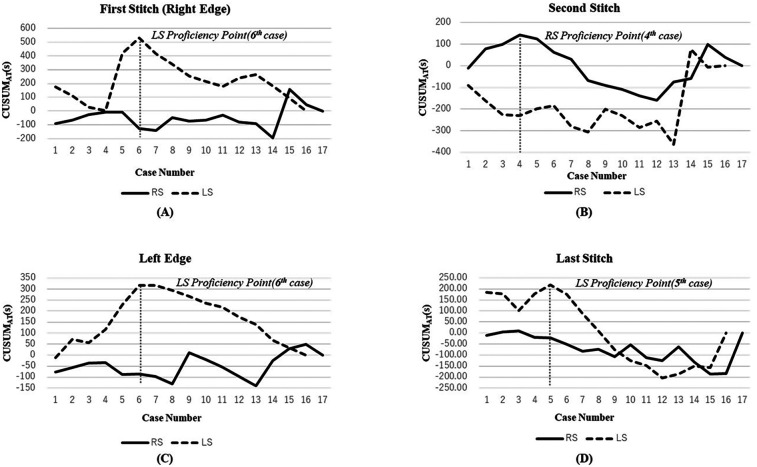
CUSUM analysis of suturing steps in hepaticojejunostomy. **(A)** First stitch (right edge); **(B)** Second stitch; **(C)** Left edge; **(D)** Last stitch. Solid lines and dashed lines represent RS and LS respectively. Vertical dashed lines indicate the transition point between Phase I (learning phase) and Phase II (proficiency phase).

The CUSUM analysis (Figure 1, Table 4) demonstrated notable differences in learning efficiency at different suturing positions:

**Table 4:** Comparison of phase Ⅰ and Ⅱ in each CUSUM learning curve.

Suture location and group	Phase Ⅰ time (s)	Phase Ⅱ time (s)	*P-*value
1st (Right edge) stitch of LS	350.5 (237.5–541)	227.5 (214.75–285.75)	0.056
Left edge stitch of LS	256.5 (173–279.5)	152.5 (148.75–164)	**≤0.01** *
2nd stitch of RS	233 (197.75–277.25)	178 (151–199)	**0.045** *
Last stitch of LS	251 (170–342.5)	170 (134–229)	0.115

Data are presented as medians (ranges).

RS, robot-assisted surgery for hepaticojejunostomy; LS, laparoscopic surgery for hepaticojejunostomy.

Phase Ⅰ represents the initial learning phase, and Phase Ⅱ represents the stabilized performance phase.

Bold values with an asterisk (*) indicate statistically significant results (*P* < 0.05).

Right-edge stitch (Figure 1A): The LS group exhibited a prolonged learning phase, stabilizing only after the sixth procedure (Phase Ⅰ: 350.5 s vs. Phase Ⅱ: 227.5 s, *P* = 0.056). This indicates that laparoscopic suturing at this position requires more practice to reach proficiency. In contrast, the RS group maintained stable performance from the first case.

Second stitch (Figure 1B): The RS group reached proficiency after four cases, showing a significant reduction in suturing time (Phase I: 233 s vs. Phase II: 178 s, *P* = 0.045).

This highlights the advantage of robotic assistance in handling complex suturing tasks.

Left-edge stitch (Figure 1C): The LS group's suturing time improved significantly after the sixth procedure (Phase Ⅰ: 256.5 s vs. Phase Ⅱ: 152.5 s, *P* ≤ 0.01). Conversely, the RS group maintained consistent precision from the first case, with minimal learning burden.

Last stitch (Figure 1D):

The LS group reached stability after five cases (Phase I: 251 s vs. Phase II: 170 s, *P* = 0.115).

The RS group demonstrated consistently low variance, with no clear “turning point”, suggesting immediate mastery of this simpler step.

These results indicate that robotic assistance accelerates proficiency in complex suturing steps while maintaining consistency across cases.

### Intraoperative comparison of RS and LS

**Figure 2:**
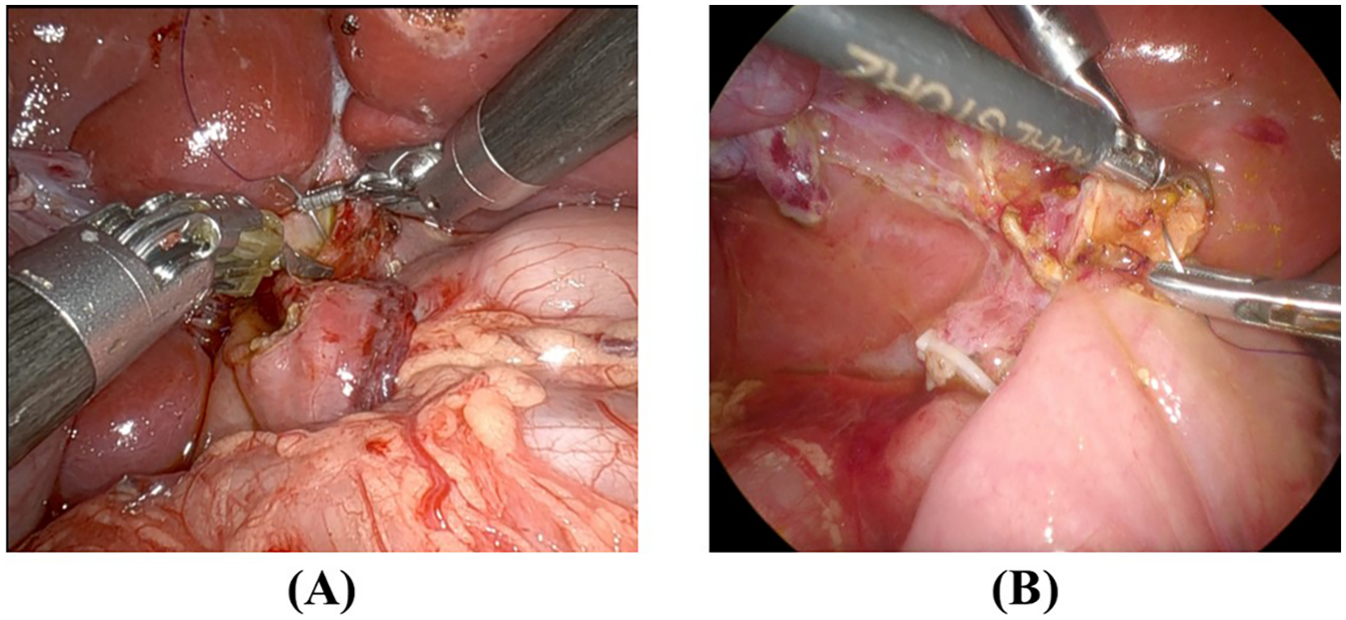
Intraoperative comparison of suturing techniques RS and LS **(A)** RS suturing at the hilar plate, demonstrating enhanced instrument articulation and stable needle control. The robotic arms provide tremor filtration and allow precise angulation. **(B)** LS suturing at the same location, requiring additional manual adjustments to maintain needle psitioning due to the limited range of motion of conventional laparoscopic instrument.

To further illustrate the differences in suturing techniques, Figure 2 presents representative intraoperative images of RS and LS hepaticojejunostomy.

RS (Figure 2A) demonstrates enhanced instrument articulation and stable needle positioning, which facilitates precise suturing near the hilar plate.

LS (Figure 2B) requires additional manual adjustments due to limited instrument maneuverability, which may contribute to increased suturing time variability.

These intraoperative observations visually support the efficiency differences observed in our suturing time analysis.

## Discussion

### Technical advantages of RS in hepaticojejunostomy

Hepaticojejunostomy is a technically demanding procedure in congenital biliary dilatation (CBD) surgery, requiring high precision within a confined surgical field. This study is the first to utilize CUSUM analysis to assess suture efficiency at specific anastomotic steps, rather than relying solely on overall anastomosis time. By providing a more granular evaluation of suturing proficiency, our findings offer new insights into the technical advantages of robot-assisted surgery (RS) over laparoscopic surgery (LS) (15–19).

We found that RS significantly reduced suture time at challenging steps, such as the second and final stitches, whereas performance at simpler sites remained comparable between the groups. The second stitch is particularly complex due to its proximity to the hilar plate, requiring precise angulation within a narrow field. The CUSUM analysis showed that the RS group achieved proficiency after just four cases at this position (Phase Ⅰ: 233 s vs. Phase Ⅱ: 178 s, *P* = 0.045), whereas the LS group demonstrated significant variability and lacked a clear turning point. This underscores the benefits of robotic articulation and tremor filtration, which enhance precision in anatomically constrained regions.

Interestingly, RS also demonstrated superior efficiency at the final stitch, despite its lower complexity. The RS group exhibited consistently low variance across all cases, with no clear “turning point,” indicating immediate mastery of this step. In contrast, the LS group required five cases to stabilize (Phase Ⅰ: 251 s vs. Phase Ⅱ: 170 s, *P* = 0.115). This finding aligns with previous studies on robot-assisted vesicourethral anastomosis, where RS outperforms LS in repetitive fine motor tasks due to its ergonomic instrument design (20).

The left-edge stitch analysis further highlighted RS's advantage in maintaining performance consistency. While the LS group exhibited a steep learning curve, stabilizing only after six cases (Phase Ⅰ: 256.5 s vs. Phase Ⅱ: 152.5 s, *P* ≤ 0.01), the RS group demonstrated stable performance from the first case onward, minimizing the learning burden. These results reinforce the hypothesis that robotic systems enhance precision while reducing operator fatigue, particularly in prolonged procedures requiring sustained dexterity.

### Per-stitch analysis enhances procedural insights

Unlike previous studies that primarily focused on total anastomosis time, our analysis of suture time per stitch provides a more precise assessment of technical performance at each step of the procedure. Total anastomosis time can be influenced by multiple factors, including anastomotic diameter and the number of stitches. In this study, the RS group had a larger anastomotic diameter and required more stitches (median 14 vs. 11, *P* < 0.001), yet maintained a consistent suture pitch (*P* = 0.846), demonstrating greater precision without compromising spacing. The reason for the larger anastomotic diameter in the RS group is not entirely clear, but it is possible that a better view of the hepatic hilum allows for a more precise lateral incision to enlarge the bile duct diameter.

Furthermore, our complication analysis showed no significant difference between RS and LS (11.8% vs. 12.4%, *P* = 0.986), suggesting that the improved efficiency with RS does not compromise patient safety. However, long-term outcomes such as anastomotic stenosis or hepatolithiasis were not systematically assessed, warranting further follow-up studies.

### Learning curve advantages of RS

Consistent with prior studies (21, 22), RS demonstrated a shorter learning phase in complex suturing steps, such as the second stitch, achieving proficiency after four cases, compared to six cases in LS. Additionally, at the left-edge and final stitches, RS maintained stable performance throughout, while LS showed greater variability during the early phase. These findings underscore the ergonomic and technical benefits of robotic systems in delicate, high-precision tasks. Our intraoperative analysis further supports these findings (Figure 2). The superior articulation of RS instruments reduced the need for repeated adjustments, allowing for greater consistency in suturing time, particularly at the second and final stitches. These observations align with our CUSUM analysis, which demonstrated a shorter learning phase and greater stability in RS compared to LS. In contrast, the increased manual adjustments required in LS may contribute to the greater variability in suturing efficiency observed in our study.

### Study limitations and future directions

Despite these promising findings, our study has limitations. First, it was a retrospective, single-surgeon study with a relatively small sample size, which may limit its generalizability. Second, this analysis was based on the suturing of a highly experienced surgeon who had already performed many other LS procedures before transitioning to RS. Therefore, it may differ from an analysis conducted on beginners. Third, blinding was not feasible during video evaluation due to the nature of the recorded surgical procedures, which could introduce potential bias in the assessment.

Future research should focus on multicenter studies with diverse surgical teams, incorporating long-term follow-up to evaluate anastomotic patency, stenosis rates, and overall clinical outcomes in pediatric hepaticojejunostomy.

## Conclusion

Our study demonstrated that RS significantly reduced suturing time compared to conventional LS, particularly at challenging steps such as the second and final stitches, while maintaining comparable complication rates. The CUSUM analysis further highlighted the shorter learning phase and greater procedural consistency of RS, suggesting an advantage in mastering complex suturing tasks.

These findings suggest that RS may be preferable for technically demanding pediatric procedures, such as hepaticojejunostomy, as it not only enhances surgical precision but also reduces the learning burden for trainees. Integrating robotic platforms into pediatric surgical training programs could accelerate skill acquisition and improve long-term proficiency.

Future multicenter studies with larger sample sizes and extended follow-up are warranted to validate these results and assess the long-term clinical outcomes, including anastomotic patency and postoperative complications.

## Data Availability

The raw data supporting the conclusions of this article will be made available by the authors, without undue reservation.
